# Long non-coding RNA UCA1 indicates an unfavorable prognosis and promotes tumorigenesis via regulating AKT/GSK-3β signaling pathway in cholangiocarcinoma

**DOI:** 10.18632/oncotarget.21884

**Published:** 2017-10-16

**Authors:** Yi Xu, Yue Yao, Kaiming Leng, Zhenglong Li, Wei Qin, Xiangyu Zhong, Pengcheng Kang, Ming Wan, Xingming Jiang, Yunfu Cui

**Affiliations:** ^1^ Department of Hepatopancreatobiliary Surgery, Second Affiliated Hospital of Harbin Medical University, Harbin, China; ^2^ The Key Laboratory of Myocardial Ischemia, Harbin Medical University, Ministry of Education, Heilongjiang, China

**Keywords:** cholangiocarcinoma, lncRNA, UCA1, EMT, AKT

## Abstract

Long non-coding RNAs (lncRNAs) have been documented to play key roles in a wide range of pathophysiological processes, including cancer initiation and progression. Recently, the aberrant expression of urothelial carcinoma associated 1 (UCA1) was observed in many types of cancers. However, its clinical relevance and exact effects in cholangiocarcinoma (CCA) remains unknown. In the present study, we aimed to investigate the clinical significance of UCA1 and evaluate its prognostic value in patients with CCA. Besides, the functional roles of UCA1 were detected both *in vitro* and *in vivo*. Moreover, potential signaling pathways were explored to clarify the molecular mechanisms underlying CCA cell proliferation. The results indicated that UCA1 transcription is enhanced in both CCA tissue samples and cell lines, and this overexpression is associated with tumor stage (*P* = 0.007), lymph node invasion (*P* = 0.027), TNM stage (*P* = 0.004) and postoperative recurrence (*P* = 0.033) of CCA patients. Besides, UCA1 could function as an independent prognostic predictor for overall survival in patients with CCA (*P* = 0.014). For the part of functional assays, knockdown of UCA1 could attenuate CCA cell growth both *in vitro* and *in vivo*. Besides, UCA1 facilitates apoptosis via Bcl-2/caspase-3 pathway. In addition, UCA1 regulates migration and invasion potential of CCA cells by affecting EMT. Furthermore, AKT/GSK-3β axis was activated to upregulate CCND1 expression due to overexpression of UCA1 in CCA. To summary, UCA1 might be a potentially useful prognostic biomarker and therapeutic target for CCA.

## INTRODUCTION

Cholangiocarcinoma (CCA) has been a heavy health burden worldwide especially in Asian countries during the past decades [1, 2]. CCA originates from biliary epithelial cells lining the biliary tree and is the second most common primary hepatic malignancy after hepatocellular carcinoma [3, 4]. Based on their localization, CCA can be divided into two groups: intrahepatic cholangiocarcinoma (ICC) and extrahepatic cholangiocarcinoma (ECC) [5]. Curative surgery, along with liver transplantation remains the only therapeutic option that offers a possibility of cure [6, 7]. Despite great advancement of surgery and systemic chemotherapy, the 5-year survival rates in CCA patients remains less than 20-40% [8]. Therefore, novel therapeutic targets by deciphering the critical molecular mechanisms accounting for the initiation and progression of CCA should be explored to better figure out the way to prevent and cure CCA.

With the progression of the human genome sequencing technology, noncoding RNAs have been regarded as key regulators of cellular transcription, which helped to gain a better understanding of the initiation and development of carcinomas [9, 10]. Long noncoding RNAs (lncRNAs), RNA molecules longer than 200 nt, act as imperative roles in both normal development and diseases including carcinomas [9]. Recently, aberrant expression of lncRNAs has been shown to be involved in multiple cancer pathogenesis and recognized as oncogenes or tumor suppressors, such as PANDAR, TUG1, H19 and etc [11–13]. However, the involvement of lncRNA in CCA is not well established.

Urothelial carcinoma associated 1 (UCA1), mapping to chromosome region of 19p13.12, was initially discovered and identified in human bladder carcinoma [14]. Some recent studies reported that UCA1 is overexpressed and has an oncogenic function in various cancers, such as hepatocellular carcinoma, breast cancer, colorectal cancer and gastric cancer [15–18]. It has been reported that UCA1 was physically associated with enhancer of zeste homolog 2 (EZH2), which suppressed p21 and E-cadherin through histone methylation on p21 and E-cadherin promoter in nucleus [19]. The ‘miRNAs sponge’ role of UCA1 was also investigated previously. In bladder cancer, UCA1 can act as a miRNA sponge and repressed the expression of miR-145 [20]. Moreover, upregulated UCA1 could promote cancer progression by regulating mTOR or Wnt signaling pathway [21, 22]. Although UCA1 has been shown to have pivotal functions in an increasing number of cancers, little is known about the expression pattern and exact role of UCA1 in CCA.

In the present study, we design the experiments to investigate the relative expression of UCA1 in CCA tissues and adjacent non-tumor tissues, and evaluated the correlation of UCA1 with clinicopathological parameters. Furthermore, cell growth, cell cycle, apoptosis, migration, invasion and epithelial-to-mesenchymal transition (EMT) were assayed to evaluate the biologic role of UCA1. The animal experiment was further introduced to validate the *in vitro* results. In addition, AKT/GSK-3β pathway was involved in the regulation of UCA1-mediated cell proliferation.

## RESULTS

### UCA1 is overexpressed in CCA tissue samples and cell lines

**Figure 1:**
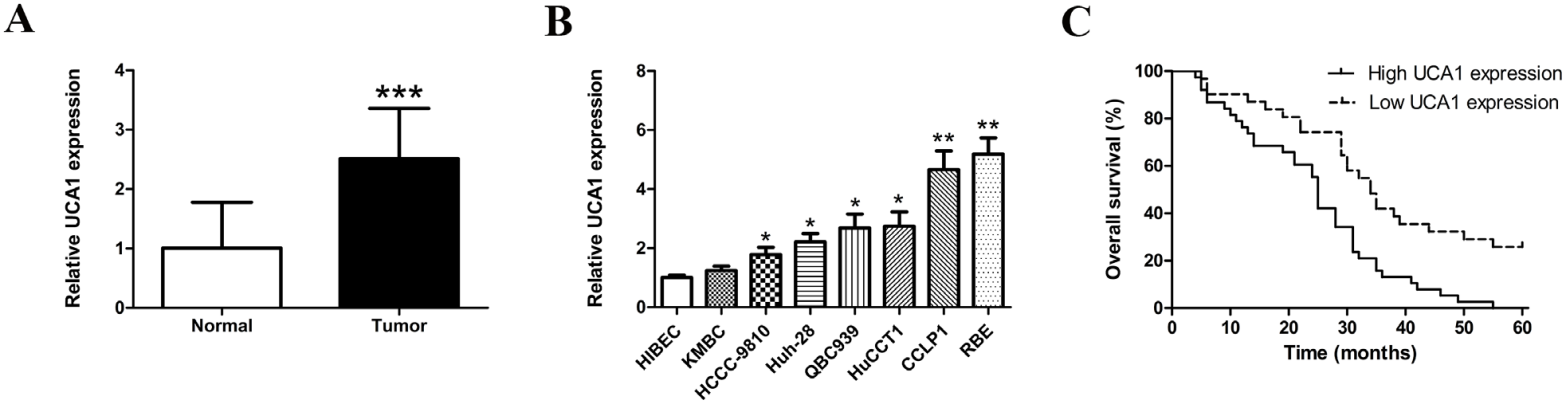
Expression levels of UCA1 in CCA samples and cell lines and its correlation with overall survival **(A)** Relative expression of UCA1 in 68 pairs of CCA tissues and corresponding normal bile duct tissues detected by qRT-PCR; **(B)** Relative expression of UCA1 in HIBEC and seven CCA cell lines detected by qRT-PCR; **(C)** Kaplan-Meier survival curves showed that overexpressed UCA1 decreased overall survival of patients with CCA. ^*^p < 0.05, ^**^p < 0.01, ^***^p < 0.001.

The expression of UCA1 in tumor tissue samples and its paired neighboring histological normal bile duct tissues of 68 patients with CCA was detected by qRT-PCR. We found that the expression level of UCA1 was significantly higher in CCA samples than that in non-cancerous counterparts (Figure 1A). Then, we detected UCA1 transcript levels in seven human CCA cell lines and human non-tumorigenic biliary epithelial cell line HIBEC. The results indicated that the expression levels of UCA1 were generally enhanced in most of the CCA cell lines (Figure 1B). Moreover, CCLP1 and RBE cells expressed the highest levels of UCA1 and were chosen for the subsequent knockdown study.

### Overexpression of UCA1 correlates with unfavorable prognosis in patients with CCA

To further explore the clinical significance of aberrant UCA1 expression, the correlation between UCA1 and CCA patients’ clinical and pathologic features were investigated. qRT-PCR analysis showed that the expression level of UCA1 in CCA tissues was 2.511 fold change of that in paired normal bile duct tissues. The transcript levels of UCA1 in all samples were then classified into low or high expression group. As shown in Table 1, the expression of UCA1 was significantly correlated with tumor stage (*P* = 0.007), lymph node invasion (*P* = 0.027), TNM stage (*P* = 0.004) and postoperative recurrence (*P* = 0.033). However, there were no obvious associations between UCA1 expression and other clinicopathological characteristics. To evaluate the prognostic value of the expression of UCA1, survival curves were analyzed by Kaplan-Meier method and compared by log-rank test. The results data demonstrated that the patients with decreased UCA1 expression had longer overall survival (*P* < 0.001, Figure 1C). The univariate Cox regression analyses of overall survival demonstrated that tumor stage (*P* = 0.020), TNM stage (*P* = 0.011), postoperative recurrence (*P*=0.021) and UCA1 expression (*P* = 0.001) were all good prognostic predictors. Moreover, UCA1 expression was confirmed as an independent prognostic indicator for overall survival in patients with CCA by multivariate analysis (*P* = 0.014, Table 2).

**Table 1:** Correlation between UCA1 expression and clinicopathological characteristics of CCA patients

Clinicopathological characteristics	No. of patients (n)	UCA1 expression		*P*-value
		High (n)	Low (n)	
Gender				
Male	30	16	14	0.807
Female	38	22	16	
Age				
<60	40	21	19	0.621
≥60	28	17	11	
Tumor site				
Intrahepatic	12	8	4	0.528
Extrahepatic	56	30	26	
Tumor stage				
T1-2	26	9	17	**0.007**
T3-4	42	29	13	
Lymph node invasion				
Present	38	26	12	**0.027**
Absent	30	12	18	
TNM stage				
I-II	21	6	15	**0.004**
III-IV	47	32	15	
Differentiation grade				
Well/moderately	24	11	13	0.307
Poorly/undifferentiated	44	27	17	
Postoperative recurrence				
Present	54	34	20	**0.033**
Absent	14	4	10	

**Table 2:** Univariate and multivariate analysis of prognostic factors for overall survival in CCA patients

Variables	Univariate analysis			Multivariate analysis		
	HR	95% CI	*P* value	HR	95% CI	*P* value
Overall Survival						
Gender (Male vs. Female)	0.955	0.573-1.592	0.860			
Age (≥60 vs. <60)	0.880	0.525-1.476	0.629			
Tumor site (Extrahepatic vs. Intrahepatic)	0.801	0.416-1.542	0.506			
Tumor stage (T3-4 vs. T1-2)	1.891	1.104-3.240	**0.020**			0.358
Lymph node invasion (Positive vs. Negative)	1.381	0.823-2.318	0.221			
TNM stage (III-IV vs. I-II)	2.088	1.182-3.689	**0.011**	1.915	1.077-3.406	**0.027**
Differentiation grade (Poorly/undifferentiated vs. Well/moderately)	1.288	0.752-2.206	0.356			
Postoperative recurrence (Present vs. Absent)	2.340	1.137-4.818	**0.021**			0.135
UCA1 expression (High vs. Low)	2.410	1.403-4.149	**0.001**	2.268	1.307-3.937	**0.014**

### UCA1 modulates cell proliferation and cell cycle in CCA cells

**Figure 2:**
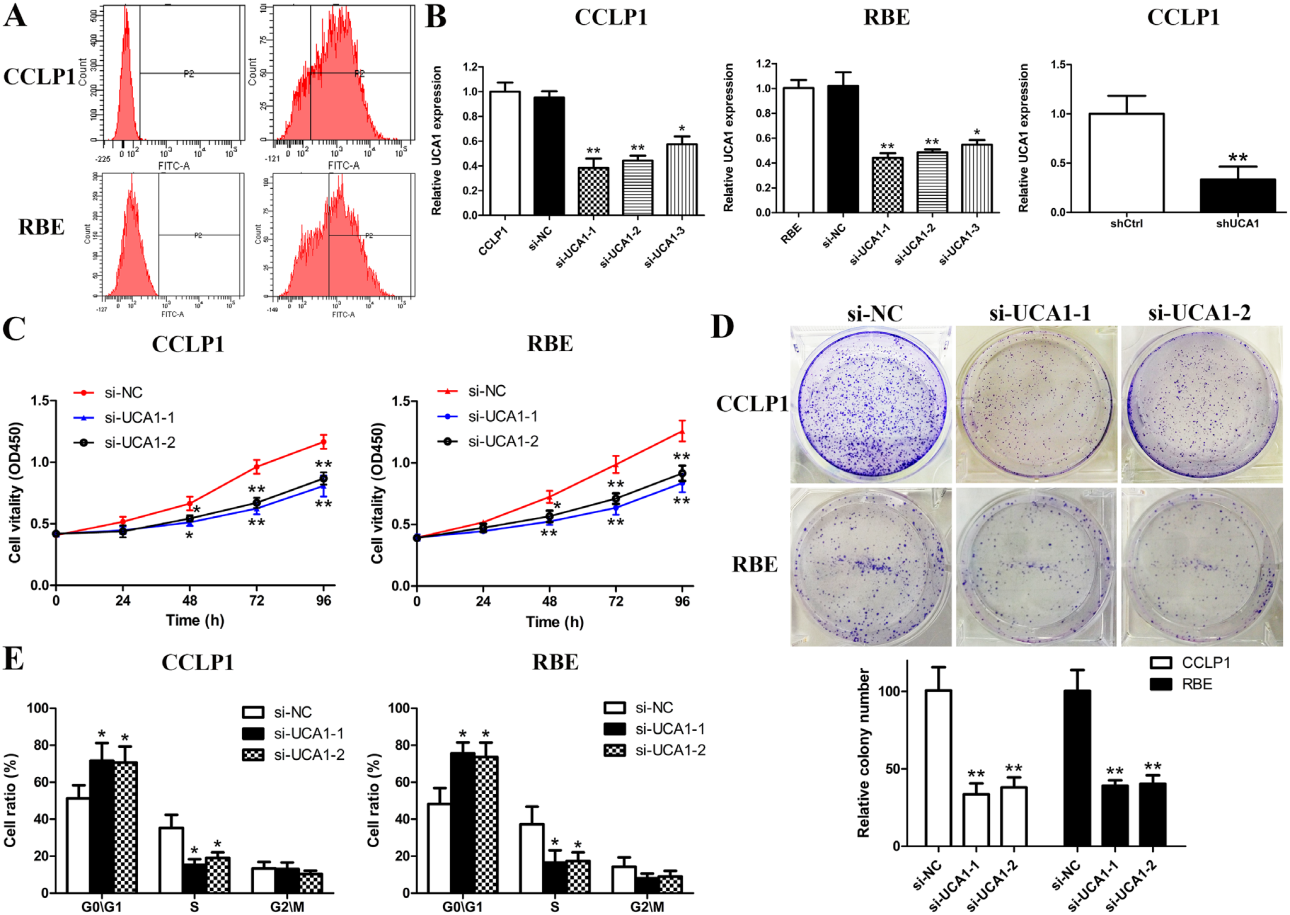
UCA1 depletion inhibits cell growth in CCA cells **(A)** Transfection efficiency detected by flow cytometry in CCLP1 and RBE cells after transfection; **(B)** Inhibition of UCA1 by transfection of UCA1 siRNAs in CCLP1 and RBE cells or shRNA in CCLP1 cells; **(C)** Cell proliferation was detected by CCK-8 assay in CCLP1 and RBE cells after transfection; **(D)** Clonogenic assay was performed to detect colony formation capacity of CCLP1 and RBE cells after transfection; **(E)** Flow cytometry analysis for cell cycle was performed to detect cell cycle distribution in CCLP1 and RBE cells after transfection. ^*^p < 0.05, ^**^p < 0.01.

The markedly overexpression of UCA1 transcription in CCA samples prompted us to investigate the possible functional roles of UCA1 in CCA. To achieve this, we ectopically knockdown UCA1 expression in CCLP1 and RBE cell lines by three different siRNAs specifically targeting UCA1. Firstly, transfection efficiency was explored by flow cyctometry and the results showed a high efficiency approximately 65%-80% in the two selected cell lines (Figure 2A). Afterwards, 48 h post-transfection, qRT-PCR was performed to detect the knockdown efficiency of UCA1. By data, si-UCA1-1 and si-UCA1-2 groups showed a stronger silencing effect on UCA1, and therefore were used for the following experiments (Figure 2B). Besides, CCLP1 cells transfected with shUCA1 also showed robust knockdown efficiency of UCA1 (Figure 2B). CCK-8 and colony formation assays demonstrated that depletion of UCA1 significantly suppressed the proliferative activity and clone formation quantity of CCLP1 and RBE cells (Figure 2C and 2D). Besides, to test whether UCA1 regulate the cell cycle distribution, flow cytometry analysis for cell cycle was performed. As indicated in Figure 2E, inhibited expression of UCA1 induced G1 cell cycle arrest in both CCLP1 and RBE cells. While the cell population in S phase was reduced remarkedly after UCA1 silenced.

### Knockdown of UCA1 promotes CCA cell apoptosis

**Figure 3:**
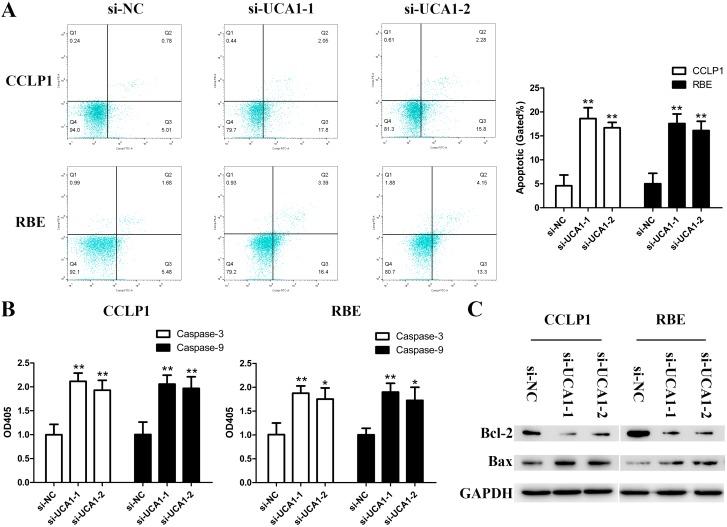
Depleted UCA1 promotes apoptosis in CCA cells **(A)** Flow cytometry analysis for apoptosis was performed to detect cell apoptosis in CCLP1 and RBE cells after transfection; **(B)** Relative expression of caspase-3 and caspase-9 in CCLP1 and RBE cells after transfection were read by microplate reader; **(C)** The protein levels of Bax and Bcl-2 in CCLP1 and RBE cells after transfection were detected by Western blot assay. ^*^p < 0.05, ^**^p < 0.01.

To assess whether the proliferative effects of UCA1 on CCA cells resulted from an alteration of cell apoptosis, flow cytometry for apoptosis analysis was performed. As shown in Figure 3A, overwhelming majority of cells were not stained positive for Annexin-V and propidium iodide in the control group. While for the two UCA1 knockdown groups, apoptotic cells increased dramatically. Meanwhile, the expression of caspase-3 and caspase-9 were both activated after UCA1 silenced (Figure 3B). It is known that Bcl-2 family proteins act as pivotal regulators of cell life and death and caspase-3, caspase-9, Bcl-2 and Bax are closely correlated with mitochondrial pathway mediated apoptosis [23]. Thus, we further explored the expression of Bcl-2 and Bax. The Western blotting data showed that down-regulated UCA1 increased apoptosis by activating the expression of Bax and suppressing Bcl-2 expression (Figure 3C).

### UCA1 depletion inhibits cell metastasis *in vitro* and affects EMT in CCA cells

**Figure 4:**
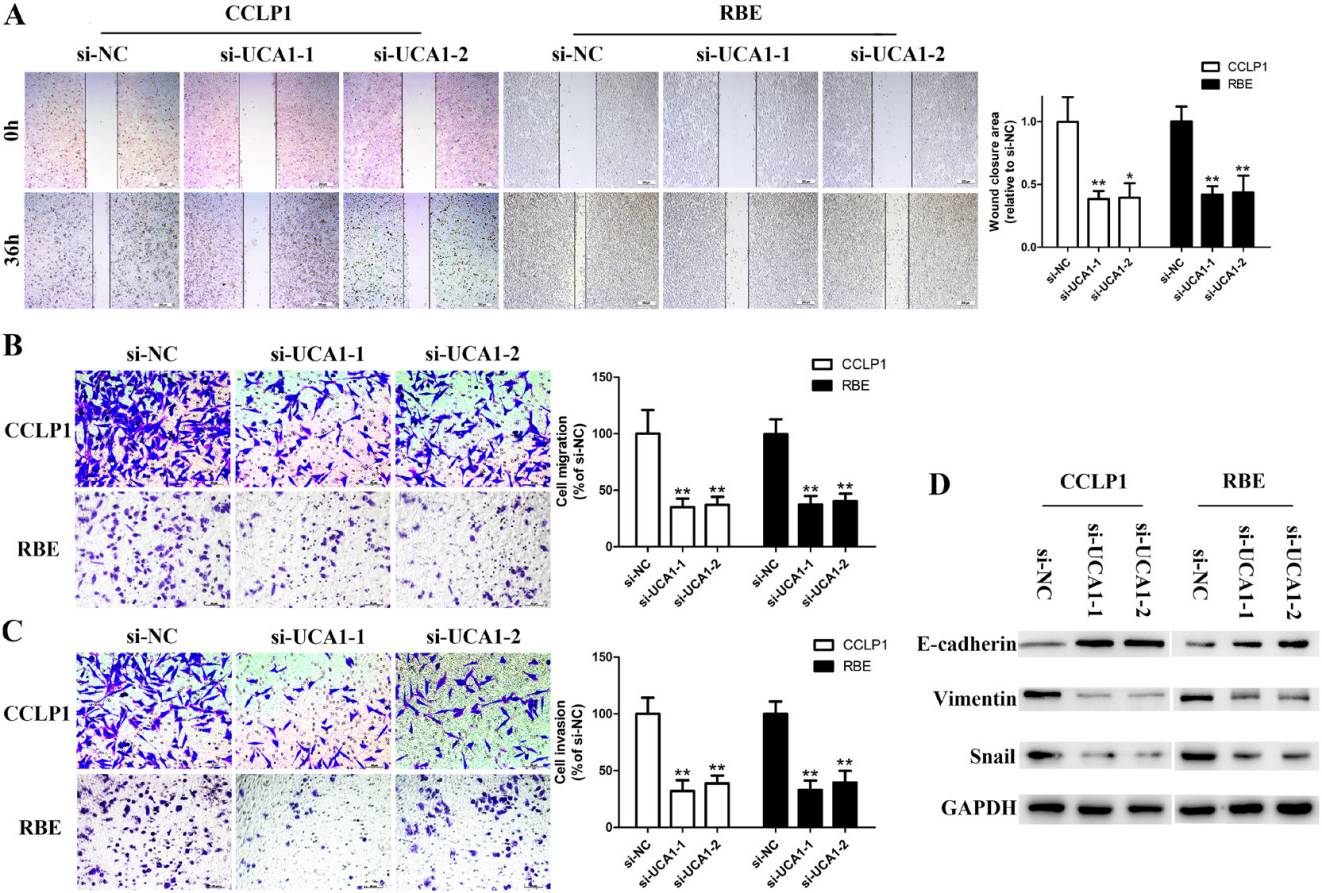
Silenced UCA1 abrogates migration and invasion capacity of CCA cells by reversing EMT **(A)** Wound healing assay was performed to detect migration ability of CCLP1 and RBE cells after transfection; **(B)** Transwell migration assay was performed to detect migration ability of CCLP1 and RBE cells after transfection; **(C)** Matrigel invasion assay was performed to detect invasion ability of CCLP1 and RBE cells after transfection; **(D)** The protein levels of E-cadherin, Vimentin and Snail in CCLP1 and RBE cells after transfection were detected by Western blot assay. ^*^P < 0.05, ^**^P < 0.01.

Given that high expression of UCA1 is associated with lymph node invasion in CCA samples, we introduced wound healing and Transwell assays to shed light on the metastasis-promoting role of UCA1 on CCA. Knockdown of UCA1 with either of the two siRNAs significantly reduced wound closure area (Figure 4A). In line with the results of wound healing assay, Transwell migration assays demonstrated that the cells passed through the membrane were dramatically decreased in the UCA1 depletion groups compared with the si-NC groups (Figure 4B). Subsequent matrigel invasion assays also showed a robust suppression of invasive potential of si-UCA1-1 and si-UCA1-2 (Figure 4C). It is known that invasion capacity of CCA cells is closely correlated with EMT process and EMT is essential prerequisites for cancer metastasis [24, 25]. By data of Western blot assay, the expression levels of Vimentin and Snail were markedly decreased. Conversely, E-cadherin was strikingly enhanced followed by UCA1 silenced (Figure 4D).

### UCA1 promoted CCA tumor formation and growth *in vivo*

**Figure 5:**
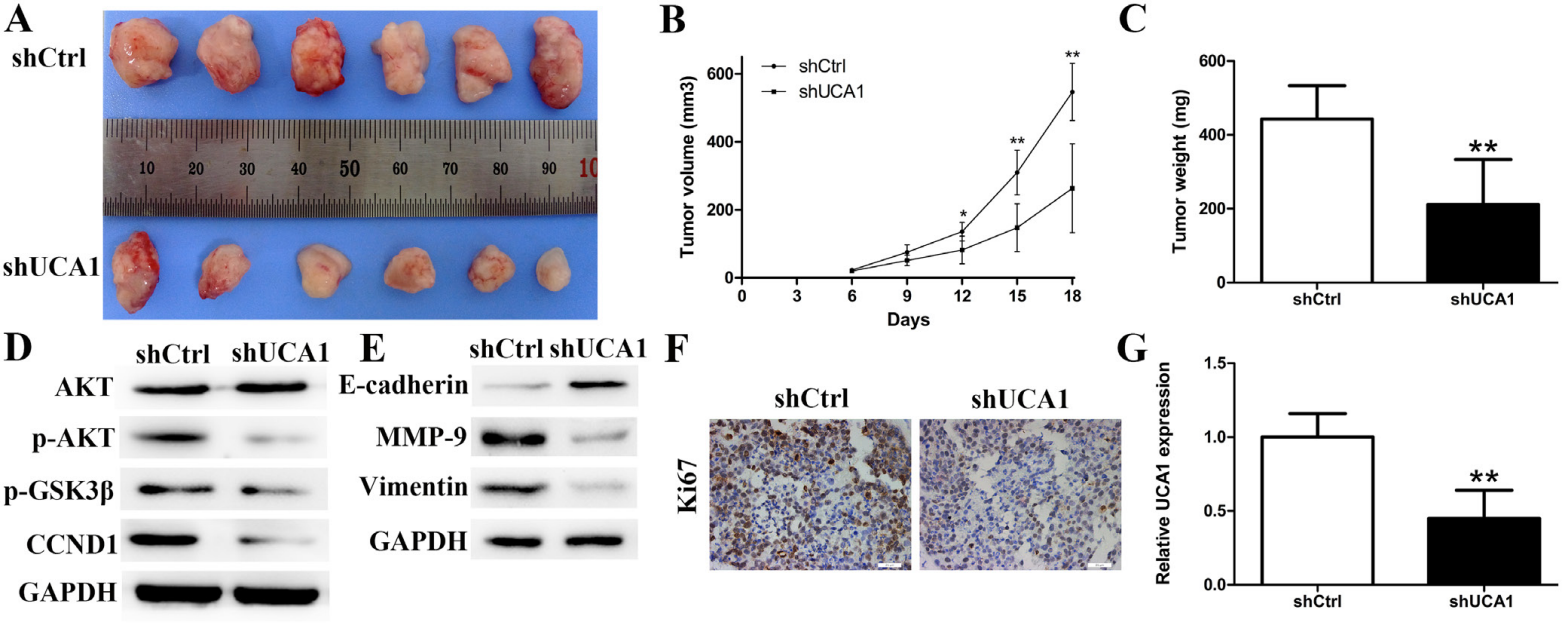
Effect of downregulated UCA1 on tumorgenesis *in vivo* **(A)** Tumors from nude mice after injection of CCLP1 cells transfected with shUCA1 or shCtrl; **(B)** Tumor volume was measured every 3 days after injection; **(C)** After 18 days of injection, tumors were removed from the nude mice and tumor weights were measured; **(D)** Relative protein levels of AKT, p-AKT, p-GSK3β and CCND1; **(E)** Relative protein levels of E-cadherin, MMP-9 and Vimentin; **(F)** The Ki67 expression and positive cell numbers was determined by immunohistochemical staining; **(G)** qRT-PCR was performed to detect the average expression of UCA1. ^*^P < 0.05, ^**^P < 0.01.

To further confirm the effects of UCA1 on tumorigenesis *in vivo*, shUCA1 CCLP1 cells and control cells were subcutaneously injected into nude mice separately. As demonstrated in Figure 5A and 5B, 18 days after inoculation, silencing of UCA1 significantly suppressed the growth of CCA xenografts. The average tumor weight in the shUCA1 group was significantly lower than that in the shCtrl group (Figure 5C). What's more, the expression of p-AKT, p-GSK3β and CCND1 were all decreased in the shUCA1 group compared to shCtrl group, which is in line with the *in vitro* results (Figure 5D). Additionally, silencing of UCA1 also reversed the EMT related markers (Figure 5E). MMP-9 has been proved to facilitate the invasion capacity of cancer cells by damaging the histological barrier around the extracellular matrix. As a result, we found MMP-9 was down-regulated after silencing of UCA1 proved by Western blot analysis (Figure 5E). Meanwhile, immunohistochemical staining indicated a decreased proliferative index Ki67 expression in shUCA1 group (Figure 5F). qRT-PCR was used to assess the expression of UCA1 in xenografts and the results showed a lower average UCA1 transcription levels in the shUCA1 group (Figure 5G).

### Upregulated UCA1 transcription activates AKT/GSK-3β/CCND1 signaling pathway

**Figure 6:**
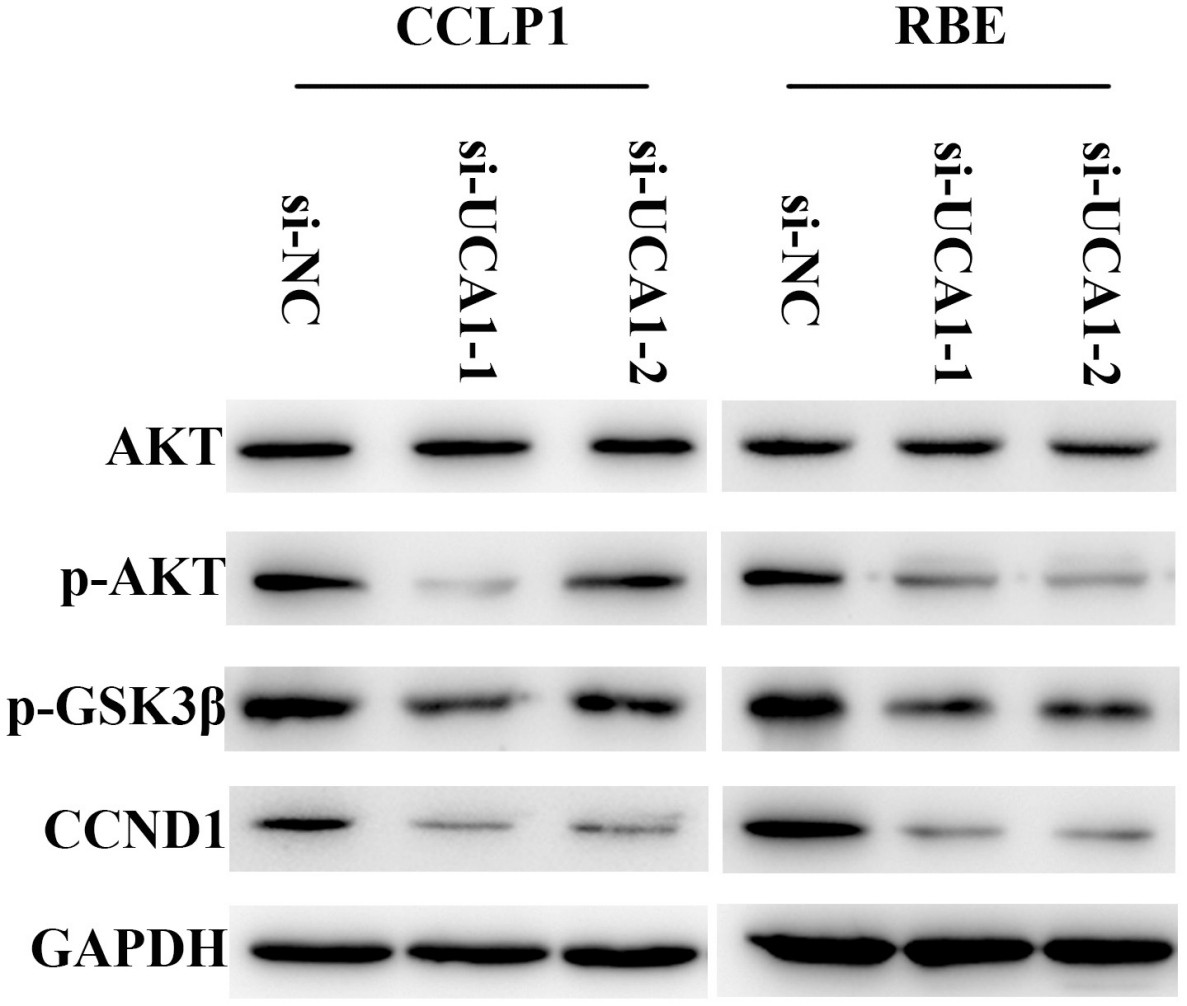
AKT/GSK-3β signaling pathway is involved in regulation of UCA1 on CCND1 expression

As we found *in vitro* and *in vivo*, UCA1 could partly inhibit CCLP1 and RBE cell proliferation. Next, we intended to identify the reasonable molecular mechanisms by which UCA1 regulates the biological features of CCLP1 and RBE cells. We detected the expression of a series of proteins likely to be affected by UCA1. The results demonstrated that knockdown of UCA1 did not affect the total expression of AKT proteins but dramatically reduced phosphorylation-AKT (p-AKT) in CCLP1 and RBE cells than that in si-NC cells. As the direct downstream of AKT signaling pathway, phosphorylation-GSK-3β (p-GSK-3β) was also down-regulated so that the suppression on CCND1 was distinctly strengthened (Figure 6). Collectively, UCA1 could activate AKT/GSK-3β/CCND1 signaling pathway to promote CCA cell cycle progression.

## DISCUSSION

Although much progression has been made in diagnostic strategies and therapeutic methods for CCA in recent years, the patients’ overall survival rates are still unfavorable due to early recurrence and metastasis. Therefore, exploring the molecular mechanisms behind the malignant biological behaviors to provide novel insights into CCA is urgently needed.

Recently, lncRNAs have gained a great deal of attention due to its abnormal expression in a wide range of carcinomas and diversiform roles in tumorigenesis, progression and metastasis [26–28]. In the present study, we intended to focus on a cancer-related lncRNA UCA1, which is overexpressed and functions as oncogenetic roles in various cancers, such as esophageal cancer, prostate cancer, renal cell carcinoma, and glioma [29–32].

In this study, the results of our investigation also demonstrated an upregulated expression of UCA1 in CCA tissues and cell lines compared with matched adjacent normal tissues and HIBEC, respectively. The upregulation of UCA1 observed in both CCA tissues and cell lines prompted us to unravel the clinical significance of UCA1 in CCA patients. A recent research demonstrated that over-expressed UCA1 contributes to poor prognosis in patients with multiple myeloma [33]. According to Zhang et al.'s study, patients with high expression of UCA1 had worse survival than those with low expression of UCA1 [34]. In addition, In agreement with most of current studies, upregulation of UCA1 in CCA was not only linked with advanced tumor stage (*P* = 0.007), high lymph node invasion rates (*P* = 0.027), late TNM stage (*P* = 0.004), and postoperative relapse (*P* = 0.033), but also could act as an independent prognostic factor for patients with CCA (*P* = 0.014).

We further explored whether UCA1 exert pivotal roles as an oncogene or simple “transcriptional junk”. Our experiments verified that knockdown of UCA1 resulted in attenuation of CCA cell proliferation by arresting cell cycle. Besides, colony formation capacity was also impaired due to UCA1 depletion. In order to understand whether the proliferative effects of UCA1 on CCLP1 and RBE cells resulted from an alteration of cell apoptosis, we conducted flow cytomery assay. Apoptosis is a form of programmed cell death critical for the development and homeostasis of multicellular organisms [35]. Among the 14 mammalian caspases identified to date, the effector caspase (caspase-3) and initiator caspase (caspase-9) play pivotal roles in the regulation of cell apoptosis. Besides, Bcl-2 family proteins function as central regulators of cell life and death. What's more, the mitochondrial and receptor-mediated apoptosis pathways are both associated with the regulation of caspase-3, caspase-9, Bax and Bcl-2 [23, 36]. In the present study, the results indicated that UCA1 serves as a defender against CCA cell apoptosis, and apoptosis-associated factors (caspase-3, -9) were both increased followed by knockdown of UCA1. Meanwhile, Bcl-2 was lost, while Bax was activated in the groups of si-UCA1-1 and si-UCA1-2. Overall, our observations documented that apoptosis-inhibiting effects of UCA1 on CCA might be achieved partly via regulating Bcl-2/caspase-3 pathway. Subsequent animal experiment further validate the proliferation-promoting effect of UCA1 on nude mice.

Overexpressed UCA1 in CCA samples is associated with positive lymphnode invasion promoted us to explore the potential influence of UCA1 on CCA cell migration and invasion. In fact, EMT is an essential prerequisite mechanism contributing to increasing cell invasion potential and driving the initiation of metastasis of cancer cells [24, 25]. Recently, lncRNAs are reported as potent regulators of tumor metastasis and EMT [37]. For example, lncRNA FOXF1-AS1 regulates non-small cell lung cancer cells metastasis via EMT [38]. For UCA1, pieces of evidence also documented that ectopic expression of UCA1 boosted cell migration and invasion via activating the vimentin and snail expression, and suppressed E-cadherin and zonula occludens-1 protein levels in gastric cancer [39]. Besides, a recent study reported that UCA1 facilitates EMT of breast cancer cells via enhancing Wnt/β-catenin signaling pathway [40]. In line with previous studies on gastric and breast cancers, the Western blotting results demonstrated a reversed EMT markers after silencing of UCA1 in both CCLP1 and RBE cells. These findings indicated that cell metastasis of CCA determined by EMT-related gene expression could be regulated by UCA1 silencing.

It has been reported that UCA1 enhances tamoxifen resistance in breast cancer cells via regulating AKT/mTOR signaling pathway [41]. In the present study, we investigated whether UCA1 correlates with AKT/GSK-3β signaling pathway. By performing Western blotting analysis, we confirmed lower expression of p-AKT and p-GSK3β in the UCA1 knockdown groups. Moreover, this inhibition further downregulated the expression of CCND1 and inhibited G1/S transition, which attenuated the CCA cell growth and cycle progression. We have noticed that UCA1 gathered both in the cytoplasm and nuclear region in renal cell carcinoma cells [31]. It probably play key roles at both transcriptional and post-transcriptional levels. Given the complex mechanisms of lncRNA UCA1 might have other molecular mechanisms, further studies are still needed.

Collectively, our findings identified an oncogenetic role of UCA1 in the tumorigenesis and development of CCA both *in vitro* and *in vivo*. Along with further study, UCA1 might be a therapeutic potential target as well as prognostic predictor for CCA.

## MATERIALS AND METHODS

### Patients and CCA cell lines

A total of CCA tissues and corresponding adjacent non-tumor tissues from 68 patients with CCA diagnosed by histopathological examination and undergoing surgery between February 2011 and January 2013 were collected. Informed consent was acquired from all patients participated in the study, and the study procedure was approved by the Ethics Review Committee of Harbin Medical University. There was no pre-operative therapy conducted in the recruited patients. The fresh tissue specimens were immediately frozen in liquid nitrogen after surgery to avoid RNA degradation. Clinical characteristics of all recruited patients were collected and shown in Table 1. HCCC-9810 and RBE were purchased from Type Culture of Chinese Academy of Sciences (Shanghai, China). The other cell lines including QBC939, Huh-28, HuCCT1, KMBC and CCLP-1 and human intrahepatic biliary epithelial cells (HIBEC) were preserved in our laboratory.

### RNA isolation and qRT-PCR

Total RNAs from tissue specimens or cells were extracted with TRIzol reagent (Invitrogen, Carlsbad, CA, USA) before converted to cDNA by using Transcriptor First Strand cDNA Synthesis Kit (Roche, Germany). Then, qRT-PCR was carried out with FastStart Universal SYBR Green Master (Roche, Germany) on a Bio-Rad Real-Time PCR System. The primer sets for UCA1 and GAPDH were used for qRT-PCR: UCA1 Forward, 5′-TTTGCCAGCCTCAGCTTAAT-3′ Reverse, 5′-TTGTCCCCATTTTCCATCAT-3′; GAPDH Forward, 5′-GGGAGCCAAAAGGGTCAT-3′ Reverse, 5′-GAGTCCTTCCACGATACCAA-3′.

### siRNAs interference and vector constructs

Small interfering RNAs (siRNAs) specifically targeting UCA1 or nonspecific scrambled siRNA were commercially obtained from GenePharma (Shanghai, China). Lipofectamine 3000 (Invitrogen) with serum-free medium was used to transfect siRNAs into cells. The target sequences of si-UCA1 are shown below: si-UCA1-1, sense, 5'-GGGCUUGGGACAUUUCACUTT-3', antisence, 5'-AGUGAAAUGUCCCAAGCCCTT-3'; si-UCA1-2, sense, 5'-GAGCCGAUCAGACAAACAATT-3', antisence, 5'-UUGUUUGUCUGAUCGGCUCTT-3'; si-UCA1-3, 5’-CCAGCCAUACAGGACCAGAUU-3’.

The LV3 (H1/GFP&Puro) vector was synthesized for the LV-shUCA1 (Ribobio, Guangzhou, China).

### Cell-proliferation analysis

The transfected CCA cells were seeded into 96-well plates with a concentration of 1500 cells in 100 μL of complete medium. Afterwards, 10 μL of cell counting kit-8 (CCK-8) reagent (Dojindo, Kumamoto, Japan) was added into each well before incubated at 37°C for 2 h. A microplate reader (Tecan, Männedorf, Switzerland) was used to detect absorbance value at 450 nm wavelength.

Clonogenic assay was also used to evaluate the colony-forming capacity of CCA cells. Single-cell suspension was plated in 6-well plates for approximately 14 days and maintained in 1640 medium supplemented with 10% fetal bovine serum (FBS). The colonies were washed with phosphate buffer solution (PBS), fixed with 4% paraformaldehyde and stained with 1% crystal violet (Beyotime, Beijing, China) before counting the visible colonies.

### Flow cytometry for cell cycle and apoptosis

Cells in each group were harvested at 48 h after transfection. For the cell cycle analysis, treated cells were trypsinized and fixed in ice-cold 70% ethanol for 12 h. Then, the cells were incubated with RNase A and propidium iodide (Beyotime, Beijing, China) with dyeing buffer for 30 min at 37°C in the dark. The cell cycle distribution was detected by flow cytometry (FACScan, BD Biosciences).

For the apoptosis analysis, cells were stained with Annexin V-fluorescein isothiocyanate and propidium iodide (BD, Biosciences, USA) following the manufacturer's directions. The apoptosis rates were analyzed by using flow cytometry (FACScan, BD Biosciences).

### Relative caspase activity determination

Relative caspase-3 and caspase-9 activity were detected at 48 h post-transfection by using Caspase-3 Activity Kit and Caspase-9 Activity Kit (Solarbio, Beijing, China). In Brief, cell proteins were extracted and added into 96-well plates with reaction buffer and substrate. The miscible liquids were maintained at 37°C for 4 h in the dark before quantified at a wavelength of 405 nm by a microplate reader (Tecan, Männedorf, Switzerland).

### Cell migration and invasion assays

To assess the effect of UCA1 siRNAs on migration of CCLP1 and RBE cells *in vitro*, transfected cells were allowed to grow to 80%-90% confluence in 6-well plates. Cell monolayers were scratched off with a sterile 200 μl pipette tube before the destroyed cells were washed twice with PBS. The cells were then cultivated in serum-free medium for 36 h at 37°C. Photographs were taken at 0 h and 36 h to measure the level of motility of each group. The migration was quantified by counting the average distance of cells that migrated towards the original wound field.

To further explore the migrative and invasive capacity of CCA cells, Transwell assays were conducted as presently described. Briefly, a total of 5.0×10^4^ cells transfected with si-UCA1 or si-NC were planted in 1640 medium in the upper chamber of Transwell unit (Costar, Washington, DC, USA) with or without 40 μL Matrigel (BD Biosciences, Franklin Lakes, NJ, USA) coating. The lower chamber was filled with complete medium. After 24 hours of incubation, the cells that had traversed the filter were fixed with paraformaldehyde and stained with crystal violet.

### Antibodies and western blot assay

The cell samples used for Western blotting were firstly lysed with RIPA lysis buffer (Beyotime, Beijing, China) containing protease inhibitors. The isolated proteins were quantified with a BCA Protein Assay Reagent Kit (Beyotime, Beijing, China). Briefly, equal amounts of proteins were subjected to separation by SDS-PAGE and transferred onto PVDF membranes (GE Healthcare, Piscataway, NJ, USA). The membranes were firstly probed with primary antibodies and then incubated with horseradish peroxidase-conjugated secondary antibody (Cell Signaling Technology, Danvers, USA). The protein bands were detected by using BeyoECL Plus Kit (Beyotime, Beijing, China). E-cadherin, Vimentin, Snail, MMP-9, Bax, Bcl-2, CCND1, GAPDH were obtained from Abcam (Cambridge, MA, USA). AKT, p-AKT, p-GSK3β were purchased from Cell Signaling Technology. (Danvers, MA, USA).

### Tumor xenografts study

CCLP1 cells (3×10^6^) transfected with shUCA1 were subcutaneously injected into either side of the flank area of 6-week-old female BALB/c nude mice (n = 6 per group). Tumor size was measured every 3 days and tumor volumes were calculated using the equation: V = 0.5×D×d^2^ (V, volume; D, longitudinal diameter; d, latitudinal diameter). After 18 days, the nude mice were euthanized and the tumors were excised. Tumors weight were measured and total RNA was isolated to detect the expression of UCA1. Proteins were extracted to detect the expression of AKT, p-AKT, p-GSK3β, CCND1, MMP-9, E-cadherin and Vimentin. All animal studies were performed under the supervision and guidelines of the Animal Care and Use Committee of Harbin Medical University.

### Immunohistochemistry study

Immunohistochemistry study was carried out on the paraffin-embedded tumor tissues from nude mice. The location and relative expression level of Ki67 protein was explored by the avidin-biotin-peroxidase method. The Ki67 primary antibody (Abcam, Cambridge, MA, USA) was used at a dilution of 1:200.

### Statistical analysis

All the statistical tests were performed by using GraphPad Prism 5.01 software (GraphPad Software, Inc., La Jolla, CA, USA) and SPSS 19.0 statistical software package (IBM, Armonk, NY, USA). Data were presented as mean ± standard deviation (SD) from at least three repeats. Overall survival rates of CCA patients were calculated according to the Kaplan-Meier analysis. Fisher's exact test was used to evaluate the association between clinicopathological features and UCA1 expression. Survival data were analyzed using univariate and multivariate Cox regression methods. To compare the significance of two groups, Student's t test was performed. A *P* value less than 0.05 was considered significant.

## Abbreviations

CCA: cholangiocarcinoma
CCK-8: cell counting kit-8
ECC: extrahepatic cholangiocarcinoma
EMT: epithelial-mesenchymal transition
EZH2: enhancer of zeste homolog 2
FBS: fetal bovine serum
HIBEC: human intrahepatic biliary epithelial cell
ICC: intrahepatic cholangiocarcinoma
lncRNA: long non-coding RNA
PBS: phosphate buffer solution
SD: standard deviation
siRNA: small interfering RNA
UCA1: urothelial carcinoma associated 1
